# *Salmonella* genomic island 1 (SGI1) reshapes the mating apparatus of IncC conjugative plasmids to promote self-propagation

**DOI:** 10.1371/journal.pgen.1006705

**Published:** 2017-03-29

**Authors:** Nicolas Carraro, Romain Durand, Nicolas Rivard, Charley Anquetil, Catherine Barrette, Malika Humbert, Vincent Burrus

**Affiliations:** Département de biologie, Faculté des sciences, Université de Sherbrooke, Sherbrooke, Québec, Canada; The University of Texas Health Science Center at Houston, UNITED STATES

## Abstract

IncC conjugative plasmids and *Salmonella* genomic island 1 (SGI1) and relatives are frequently associated with multidrug resistance of clinical isolates of pathogenic *Enterobacteriaceae*. SGI1 is specifically mobilized in *trans* by IncA and IncC plasmids (commonly referred to as A/C plasmids) following its excision from the chromosome, an event triggered by the transcriptional activator complex AcaCD encoded by these helper plasmids. Although SGI1 is not self-transmissible, it carries three genes, *traN*_S_, *traH*_S_ and *traG*_S_, coding for distant homologs of the predicted mating pore subunits TraN_C_, TraH_C_ and TraG_C_, respectively, encoded by A/C plasmids. Here we investigated the regulation of *traN*_S_ and *traHG*_S_ and the role of these three genes in the transmissibility of SGI1. Transcriptional fusion of the promoter sequences of *traN*_S_ and *traHG*_S_ to the reporter gene *lacZ* confirmed that expression of these genes is inducible by AcaCD. Mating experiments using combinations of deletion mutants of SGI1 and the helper IncC plasmid pVCR94 revealed complex interactions between these two mobile genetic elements. Whereas *traN*_C_ and *traHG*_C_ are essential for IncC plasmid transfer, SGI1 could rescue null mutants of each individual gene revealing that TraN_S_, TraH_S_ and TraG_S_ are functional proteins. Complementation assays of individual *tra*_C_ and *tra*_S_ mutants showed that not only do TraN_S_/H_S_/G_S_ replace TraN_C_/H_C_/G_C_ in the mating pore encoded by IncC plasmids but also that *traG*_S_ and *traH*_S_ are both required for SGI1 optimal transfer. In fact, remodeling of the IncC-encoded mating pore by SGI1 was found to be essential to enhance transfer rate of SGI1 over the helper plasmid. Furthermore, *traG*_S_ was found to be crucial to allow DNA transfer between cells bearing IncC helper plasmids, thereby suggesting that by remodeling the mating pore SGI1 disables an IncC-encoded entry exclusion mechanism. Hence *tra*_S_ genes facilitate the invasion by SGI1 of cell populations bearing IncC plasmids.

## Author summary

Acquisition and dissemination of multidrug resistance genes among *Enterobacteriaceae* is in part driven by IncA and IncC (A/C) conjugative plasmids and *Salmonella* genomic island 1 (SGI1). Although unrelated, SGI1 relies on the self-transmissible A/C plasmids to disseminate within bacterial populations. The mechanisms allowing SGI1 to hijack the mating apparatus synthesized by A/C plasmids have not been previously established. Here, we show that IncC plasmids trigger the expression of three SGI1-borne genes that code for functional mating pore subunits distantly related to those encoded by the IncC helper plasmids. Our results indicate that these subunits alter the mating pore encoded by IncC plasmids to ensure optimal transfer of SGI1 and promote SGI1 dissemination in cell populations harboring IncC plasmids. Apart from SGI1 and relatives, documented mobilizable genomic islands are not known to code for mating pore components, possibly because of redundancy with those encoded by helper conjugative elements. Instead they usually code for mobilization proteins such as a relaxase and auxiliary factors involved in DNA recognition, processing and docking to the mating pore encoded by their helper conjugative element. From an ecological and epidemiological perspective, the strategy used by SGI1 likely confers a strong competitive advantage to SGI1 over IncC plasmids in clinical settings and could account for the high prevalence of SGI1 and relatives in multidrug-resistant *Salmonella enterica* and *Proteus mirabilis*.

## Introduction

Conjugation is a nearly ubiquitous mechanism of horizontal gene transfer in bacteria, allowing the exchange of the largest number of genes per transfer event, often across taxonomical barriers [1–3]. In Gram-negative and most Gram-positive bacteria, conjugation is mediated by a complex nano-machine called type IV secretion system (T4SS). Conjugative T4SSs are multiprotein complexes that span the cell envelope and translocate DNA substrates from a donor to a recipient cell [4]. Conjugative plasmids and integrative and conjugative elements (ICEs) code for T4SS to promote their own dissemination by conjugation. These self-transmissible mobile genetic elements often bear a gene cargo of metal and antibiotic resistance genes, virulence determinants and other traits with potential selective advantages for the bacterial host [2,5–7].

**Fig 1:**
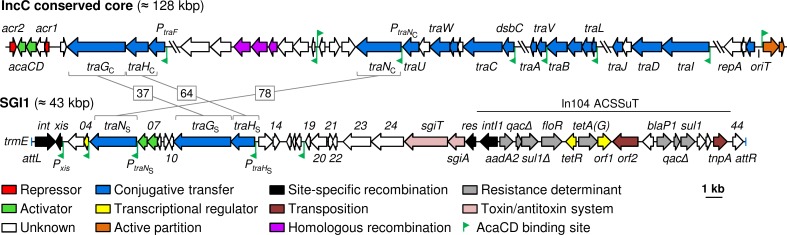
Linear schematic representation of the core sequence of IncC plasmids and of *Salmonella* genomic island 1 (SGI1). The position and orientation of open reading frames (ORFs) are indicated by arrowed boxes. Colors depict the function deduced from functional analyses and BLAST comparisons. AcaCD binding sites are represented by green angled arrows. SGI1 is flanked by the *attL* (vertical blue line on the left) and *attR* (vertical blue line on the right) attachment sites when integrated into the 3’ end of the *trmE* gene in the chromosome of *Salmonella enterica* serovar Typhimurium DT104. ACSSuT, resistance to ampicillin, chloramphenicol, streptomycin/spectinomycin, sulfamethoxazole and tetracycline.

Conjugative plasmids of the incompatibility group C (IncC) have been found in a broad range of *Enterobacteriaceae* and in *Vibrio cholerae* in which they can replicate and efficiently transfer [8–11]. IncC plasmids are closely related to IncA plasmids, and together are collectively referred to as A/C plasmids [8]. IncC plasmids are often recovered from clinical isolates of major pathogenic bacteria to which they confer resistance against multiple drugs, including last-resort antibiotics such as carbapenems [8,12,13]. IncC plasmids share a common scaffold of genes necessary for their replication, stability, conjugative transfer and regulation (Fig 1) [8,14]. Expression of the conjugative transfer genes of IncC plasmids is controlled by repressors Acr1 and Acr2 [15]. These two transcriptional repressors control the transcription of an operon containing *acaC* and *acaD* that code for the two subunits of the master activator AcaCD [15,16]. In IncC plasmids, AcaCD specifically binds to and activates 18 promoters that drive the expression of multiple genes and operons, most of which are of unknown function [15]. A third of these promoters drive the expression of *tra* genes coding for F-type T4SS assembly (*traLEKB*, *traVA*, *dsbC/traC/trhF*/*traWU*, *traFHG*), mating pair stabilization (*traN*), and the relaxase and type IV coupling protein (*traIDJ*) necessary for conjugative transfer (Fig 1) [17,18].

AcaCD also activates the expression of genes carried by unrelated mobilizable genomic islands (MGIs), thereby triggering their excision from the chromosome and their IncC-dependent dissemination into new bacterial hosts. Such MGIs include MGI*Vmi*1 from *Vibrio mimicus*, MGI*Vch*Hai6 from *Vibrio cholerae* and *Salmonella* genomic island 1 (SGI1) from *Salmonella enterica* serovar Typhimurium DT104 [15,19–22]. SGI1 and its multiple variants are frequently found in *S*. *enterica* and *Proteus mirabilis* clinical isolates [23–25]. SGI1 and relatives all share a common 26-kb core region disrupted by a complex class 1 integron conferring multidrug resistance (Fig 1) [23,26,27].

SGI1 is thought to hijack the conjugative apparatus encoded by IncA and IncC plasmids to transfer to a new host cell by a mechanism that remains unknown as no origin of transfer (*oriT*) or mobilization protein such as a relaxase has been identified in SGI1 to date [28]. Remarkably, SGI1 was reported to be mobilized at a much higher rate than MGI*Vmi*1 and MGI*Vch*Hai6 (~3 logs higher) when mobilized by the same IncC plasmid, even outperforming the helper plasmid by 10 fold [15,20]. Unlike MGI*Vmi*1 and MGI*Vch*Hai6, the core region conserved in SGI1-related elements codes for two putative T4SS subunits, TraG_S_ and TraH_S_, as well as a putative mating pair stabilization protein, TraN_S_, that are distantly related to the counterparts TraG_C_ (Vcrx144), TraH_C_ (Vcrx143) and TraN_C_ (Vcrx084) encoded by IncC plasmids (Fig 1, Table 1) [18,29–33]. While these observations suggest that the putative T4SS subunits encoded by SGI1 could be involved in SGI1 spread, they have been shown to be dispensable for its mobilization [28]. Like *xis*, a gene coding for the recombination directionality factor Xis that facilitates the excision of SGI1 from the chromosome, expression of the three *tra* genes of SGI1 has recently been reported to be under the control of AcaCD (Fig 1) [15,34,35]. This raises the question of the functional role of the putative *tra* genes of SGI1.

**Table 1:** Protein homologs encoded by IncC plasmids and SGI1.

Name	Length (aa)	Identity	Similarity	Coverage	Signal peptide	Pfam domain	Predicted function
TraN_C_/TraN_S_	933/920	78%	88%	97%	yes	PF06986	Mating pair stabilization, adhesin
TraH_C_/TraH_S_	478/475	64%	78%	92%	yes	PF06122	Mating apparatus formation/stabilization
TraG_C_/TraG_S_	1205/1135	37%	57%	99%	no	PF07916	Mating apparatus stabilization, entry exclusion

In this study, we investigated whether SGI1 could alter the mating pore encoded by IncC plasmids to enhance its own transfer. First, we confirmed that the genes *traN*_S_, and *traHG*_S_ of SGI1 are under AcaCD control and that this cluster of *tra* genes is important for SGI1 mobilization. Using combinations of deletion mutants and complementation assays, we explored the role of each Tra_C_/Tra_S_ subunit on the formation of the mating pore and its proficiency to mediate transfer of SGI1 and/or the helper IncC plasmid. Finally, we demonstrated that substitution of the TraG subunit enables SGI1 to escape an entry exclusion mechanism encoded by IncC plasmids.

## Results

### SGI1 *tra*_S_ genes are induced by the master activator of transfer of IncC plasmids

Expression of *traN*_C_ and *traHG*_C_ of IncC plasmids is activated by AcaCD, which recognizes a specific AcaCD box upstream of the -35 sequence in the promoters *P*_*traN*c_ and *P*_*traF*_, respectively (Fig 1) [15]. Two AcaCD boxes were also predicted in intergenic sequences upstream of *traN*_S_ and *traHG*_S_. To test whether expression of SGI1 *tra* genes is AcaCD-dependent, promoters of *traN*_S_ (*P*_*traN*s_) and *traHG*_S_ (*P*_*traH*s_) were cloned upstream of a promoterless *lacZ* gene and their activity was measured by β-galactosidase assays. Controls included the constitutive promoter *P*_*int*_ and the AcaCD-inducible promoter *P*_*xis*_ that drive the expression of the integrase- and Xis-coding genes of SGI1, respectively.

**Fig 2:**
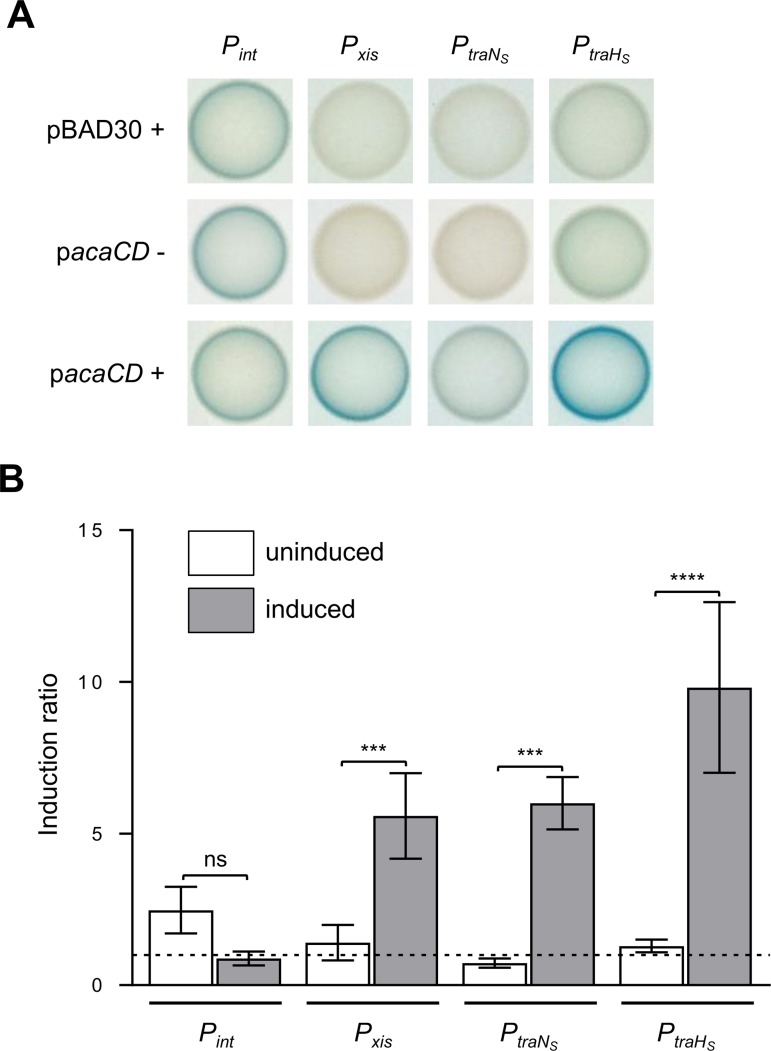
Regulation of expression of *int*, *xis*, *traN*_S_ and *traHG*_S_ of SGI1. (A) Activity of *P*_*int*_, *P*_*xis*_, *P*_*traN*s_ and *P*_*traH*s_ was monitored from single-copy, chromosomally integrated *lacZ* transcriptional fusions in *E*. *coli* BW25113 Nx. Colorimetric assays of β-galactosidase activity were carried out on LB medium supplemented with (+) or without (-) arabinose to express *acaCD* from *P*_*BAD*_ on p*acaCD*. (B) Induction of *P*_*int*_, *P*_*xis*_, *P*_*traN*s_ and *P*_*traH*s_ in response to AcaCD. β-galactosidase assays were carried out using the same strains as in panel A. Ratios between normalized OD_420_ values in the arabinose-induced over non-induced p*acaCD*, and non-induced p*acaCD* over arabinose-induced empty pBAD30 vector are shown. The bars represent the mean and standard deviation values obtained from at least three independent experiments. Statistical analyses were performed using the one-way ANOVA followed by Sidak’s post-test to compare each induction ratio to its corresponding control. Statistical significance is indicated as followed: ****, *P* < 0.0001; ***, *P* < 0.001; ns, not significant.

Activity of *P*_*traN*s_ was below the level of detection in absence of AcaCD, whereas in the same condition, *P*_*traH*s_ exhibited a weak yet detectable constitutive activity that remained weaker than the constitutive expression of *P*_*int*_ (Fig 2A). AcaCD increased the activity of *P*_*traN*s_ and *P*_*traH*s_ by 6 and 10 fold, respectively, as observed for *P*_*xis*_ (Fig 2A and 2B). Therefore, like their IncC counterparts, expression of SGI1-borne *tra*_S_ genes is directly stimulated by AcaCD, suggesting that TraN_S_, TraH_S_ and TraG_S_ of SGI1 are produced alongside with TraN_C_, TraH_C_ and TraG_C_ of IncC plasmids. Thus, the SGI1-coded Tra_S_ subunits might complement or even compete with those encoded by IncC plasmids and lead to the synthesis of a hybrid T4SS with altered properties.

### TraN_C_, TraH_C_ and TraG_C_ are essential for IncC plasmid transfer

**Fig 3:**
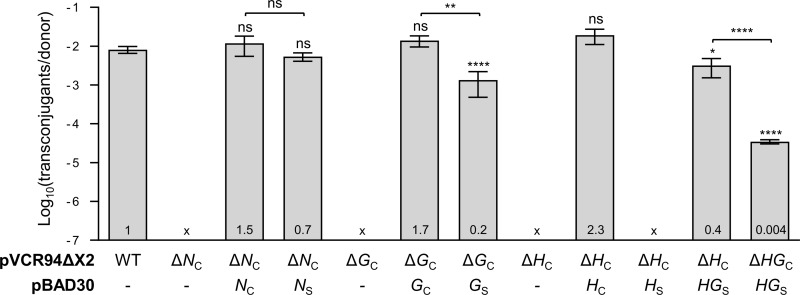
Role of TraN/G/H_C_ on conjugative transfer of IncC plasmids. Effect of *traN*_C_, *traG*_C_ and *traH*_C_ on conjugative transfer of pVCR94ΔX2. Conjugation assays were carried out using *E*. *coli* BW25113 Nx containing the indicated elements as donor strains and *E*. *coli* CAG18439 (Tc) as the recipient strain. Wild-type (WT) or derivative mutants of pVCR94ΔX2 as well as the gene expressed from pBAD30 are indicated below each graph; (-) indicates that the plasmid is not present in the donor cell. For clarity, gene names *traX*_Y_ were shortened *X*_Y._ Transfer frequencies are expressed as the number of transconjugants per Nx^R^ Kn^R^ Sp^R^ donor CFUs and the ratio of transfer frequencies relative to WT is indicated at the base of each bar. The bars represent the mean and standard deviation values obtained from at least three independent experiments. “x” indicates that the frequency of transfer was below the detection limit (<10^−7^). Statistical analyses were carried out on the logarithm of the values using the one-way ANOVA with Tukey’s multiple comparison test. Statistical significance is indicated as followed: ****, *P* < 0.0001; ***, *P* < 0.001; **, *P* < 0.01; *, *P* < 0.05; ns, not significant.

While predicted to be part of the mating apparatus of IncC plasmids, whether TraN_C_, TraH_C_ or TraG_C_ are necessary for conjugative transfer has not yet been established [8]. To investigate this, we carried out mating experiments using a set of deletion mutants of pVCR94ΔX2 (Su Sp) as well as complementation assays aimed at evaluating their importance for conjugative transfer. Individual deletion of *traN*_C_, *traH*_C_ or *traG*_C_ completely abolished conjugative transfer of pVCR94ΔX2 (Fig 3). *Trans*-complementation of each deletion mutant by expressing the missing gene from the medium-copy plasmid pBAD30 under the control of the arabinose-inducible *P*_*BAD*_ promoter restored pVCR94ΔX2 transfer to wild-type level, thereby confirming that the mutations were non-polar (Fig 3). Therefore, the predicted T4SS subunits TraN_C_, TraG_C_ and TraH_C_ are essential for conjugative transfer of IncC plasmids.

### The *tra*_S_ gene cluster is important for SGI1 mobilization

To facilitate further investigations on SGI1, we substituted its multidrug resistance locus In104 with a kanamycin-resistance (Kn) cassette, while preserving the core genes conserved in all SGI1-like elements (Fig 1). Resulting SGI1ΔIn104 (Kn) was then used with pVCR94ΔX2 to investigate the role of *traN*_S_ and *traHG*_S_, and of their respective IncC-borne homologs in the dissemination of SGI1 and IncC plasmids.

As shown in Fig 2, expression of *traN*_S_, *traH*_S_ and *traG*_S_ in SGI1 is triggered by AcaCD of IncC plasmids. Nevertheless, whether these genes code for proteins able to contribute to the formation of a functional T4SS was unclear. Kiss *et al*. [28] reported that mobilization of a natural SGI1 variant lacking ~10 kb (from 3,610 to 13,537 bp) including open reading frames from *traN*_S_ to *traH*_S_ was not significantly impacted by the deletion. This suggests the region encompassing *traN*_S_ to *traH*_S_ does not contain indispensable functions for SGI1 mobilization. To verify this, we constructed a Δ*tra*_S_ mutant of SGI1ΔIn104 lacking the whole *traN*_S_ to *traH*_S_ region and compared the mobilization by pVCR94ΔX2 of SGI1ΔIn104 and its Δ*tra*_S_ mutant. Unlike previously reported [28], we observed that Δ*tra*_S_ led to ~4,000-fold decrease of transfer (2.27 ± 1.42 for SGI1ΔIn104 vs 5.46×10^−4^ ± 1.20×10^−4^ for its Δ*tra*_S_ mutant), thereby suggesting that genes included in the *tra*_S_ gene cluster are important for SGI1 mobilization.

### SGI1 complements a defective IncC-encoded T4SS with functional subunits

**Fig 4:**
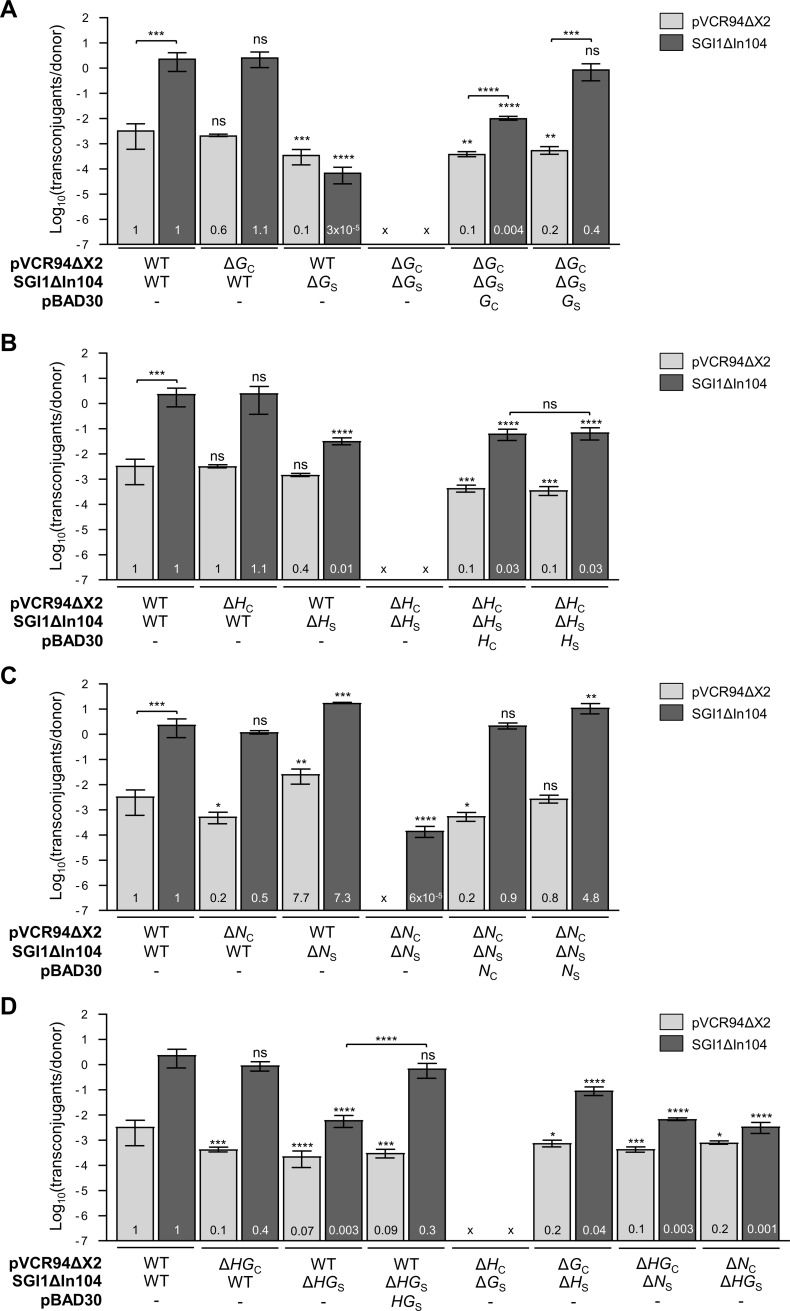
Role of *tra*_C_ and *tra*_S_ genes on conjugative transfer of IncC plasmids and SGI1. (A) Effect of *traG*_C_ and *traG*_S_ on conjugative transfer of pVCR94ΔX2 (light gray bars) and SGI1ΔIn104 (dark gray bars). (B) Effect of *traH*_C_ and *traH*_S_. (C) Effect of *traN*_C_ and *traN*_S_. (D) Effect of combinatory deletions. For details, refer to legend of Fig 3. Two one-way ANOVA with Tukey’s multiple comparison test were carried out separately for each element. The unpaired *t*-test (two-tailed) was performed to compare the bars of different elements. WT frequencies of transfer for both elements come from a single set of experimental replicates but are displayed throughout panels A to D as a reference in each statistical analysis.

We took advantage of the non-transmissible *traN*_C_, *traH*_C_ and *traG*_C_ null mutants of pVCR94ΔX2 that likely produce a non-functional mating apparatus to test whether SGI1ΔIn104 could rescue such mutants. If the putative T4SS subunits encoded by SGI1ΔIn104 can replace the ones missing in the T4SS encoded by pVCR94ΔX2, transmissibility of the plasmid should be restored. Remarkably, SGI1ΔIn104 could restore conjugative transfer of each individual mutant to levels that were comparable to wild-type pVCR94ΔX2, while also allowing its own transfer (second pair of bars in Fig 4A, 4B and 4C). These results indicate that SGI1 produces functional mating pore components that can replace the missing corresponding parts in the IncC T4SS.

To confirm this hypothesis, *trans*-complementation of the *traN*_C_, *traG*_C_ and *traH*_C_ null mutants of pVCR94ΔX2 was carried out by providing donor cells with *traN*_S_, *traG*_S_ and *traH*_S_, respectively, expressed from pBAD30. Expression of *traN*_S_ restored conjugative transfer of pVCR94ΔX2 Δ*traN*_C_ to wild-type level (Fig 3). Surprisingly, despite its strong divergence from TraG_C_ (Table 1), TraG_S_ restored conjugative transfer of pVCR94ΔX2 Δ*traG*_C_ with only a mere 5-fold reduction compared to wild-type (Fig 3). In contrast, although TraH_C_ and TraH_S_ share 64% identity, expression of *traH*_S_ failed to restore conjugative transfer of pVCR94ΔX2 Δ*traH*_C_ (Fig 3). This latter observation was puzzling because SGI1ΔIn104 could complement the *traH*_C_ null mutant of pVCR94ΔX2.

### TraG_S_ is required for optimal SGI1 transfer

As shown above, TraG_S_ is a functional substitute for TraG_C_ as it restored transfer of pVCR94ΔX2 Δ*traG*_C_ (Fig 3). In contrast, when the reciprocal experiment was carried out, we found that TraG_C_ is a poor substitute for TraG_S_ for mediating transfer of SGI1ΔIn104. While SGI1ΔIn104 transfer was unaffected by the Δ*traG*_C_ mutation in pVCR94ΔX2 (Fig 4A), SGI1ΔIn104 Δ*traG*_S_ transfer was strongly reduced despite the presence of wild-type pVCR94ΔX2. Taken together, these results suggest that TraG_S_ is required for optimal transfer of SGI1 whereas efficient transmissibility of IncC plasmids can be mediated by TraG_C_ and/or TraG_S_ (Figs 3 and 4A).

Combination of both *traG*_C_ and *traG*_S_ mutations completely abolished transfer of both elements, confirming the key role of this T4SS component (Fig 4A and S1A Fig). In this context, overexpression of either *traG*_C_ or *traG*_S_ nearly restored full transfer of pVCR94ΔX2 Δ*traG*_C_. However, *traG*_C_ was inefficient at complementing SGI1ΔIn104 Δ*traG*_S_ and resulted in a 250-fold decrease of transfer compared to SGI1ΔIn104 (Fig 4A). In contrast, providing *traG*_S_ in *trans* fully restored transfer of SGI1ΔIn104 Δ*traG*_S_. Therefore, *traG*_S_ seems to be a key factor for enhancing the transmissibility of SGI1 relatively to IncC plasmids.

### TraH_S_ is specifically required with TraG_S_ for SGI1 optimal transfer

Overexpression of *traH*_S_ was unable to complement a *traH*_C_ null mutant of pVCR94ΔX2, whereas wild-type SGI1ΔIn104 rescued pVCR94ΔX2 Δ*traH*_C_ (Figs 3 and 4B). As *traH*_S_ and *traG*_S_ seem to be part of the same operon in SGI1, like their homologs *traH*_C_ and *traG*_C_ in IncC plasmids (Fig 1), we suspected that the products of these genes might be interacting partners in the T4SS [14,15]. Substitution of a cognate partner within a pair of interacting proteins by a homologous protein encoded by another element is likely to impair these interactions and affect the functionality of the resulting hybrid mating pore. To test this hypothesis, complementation assays were performed using p*traHG*_S_ to coexpress both *traH*_S_ and *traG*_S_ in cells bearing either pVCR94ΔX2 Δ*traH*_C_ or pVCR94ΔX2 Δ*traHG*_C_ (Fig 3). In both cases, conjugative transfer of pVCR94ΔX2 was partially restored, thereby confirming that TraG_S_ and TraH_S_ work together within the mating pore. Together with the lack of complementation of pVCR94ΔX2 Δ*traH*_C_ by p*traH*_S_, this latter observation likely reflects the inability of the SGI1-encoded TraH_S_ subunit to interact with the IncC plasmid-encoded TraG_C_ subunit to form a functional T4SS.

Although SGI1ΔIn104 Δ*traH*_S_ combined with pVCR94ΔX2 did not prevent the formation of a functional mating pore since pVCR94ΔX2 transferred at wild-type level, we observed that transfer of SGI1ΔIn104 Δ*traH*_S_ was reduced by 100 fold (Fig 4B). Because TraG_S_ is required for optimal transfer of SGI1ΔIn104 (Fig 4A), this observation suggests that although TraH_S_ does not work in association with TraG_C_, TraH_C_ associated with TraG_S_ could form a functional mating pore.

To investigate further these interactions, we combined pVCR94ΔX2 and SGI1ΔIn104 mutants, as well as expression of Tra_C_/Tra_S_ subunits from pBAD30 vectors in conjugative transfer experiments. Neither element transferred when the *traH*_C_*/traH*_S_ combination of mutants was used (Fig 4B and S1B Fig). However, complementation by providing either *traH*_C_ or *traH*_S_ in *trans* partially restored transfer of both elements. When pVCR94ΔX2 Δ*traH*_C_ was combined with SGI1ΔIn104 Δ*traG*_S_, no transfer was detected for either element (Fig 4D), thereby confirming that TraG_C_ and TraH_S_ are incompatible and unable to form a functional T4SS. In contrast, the reciprocal association of pVCR94ΔX2 Δ*traG*_C_ with SGI1ΔIn104 Δ*traH*_S_ allowed transfer of both elements to near wild-type levels, thereby confirming that association of TraH_C_ with TraG_S_ can sustain formation of a functional and efficient hybrid T4SS (Fig 4B).

Altogether, these results revealed that while SGI1 can use a mating pore entirely encoded by IncC plasmids, the expression and association of TraH_S_ with TraG_S_ are necessary for its optimal transfer, which largely surpasses the transfer rate of the helper plasmid.

### TraN_C_ and TraN_S_ proteins are exchangeable

Additional experiments were performed to assess the impact of *traN*_C_ and *traN*_S_ on the transfer ability of pVCR94ΔX2 and SGI1. Transfer assays using a donor bearing pVCR94ΔX2 and SGI1ΔIn104 Δ*traN*_S_ revealed that *traN*_S_ was dispensable for SGI1 transfer if the IncC helper plasmid provided TraN_C_ (Fig 4C). Combination of Δ*traN*_C_ and Δ*traN*_S_ mutations abolished pVCR94ΔX2 transfer, although it allowed residual transfer of SGI1ΔIn104 Δ*traN*_S_ (>4 logs below SGI1ΔIn104 level). Complementation of both mutations using either p*traN*_C_ or p*traN*_S_ restored the transmissibility of both elements (Fig 4C and S1C Fig).

### TraN_S_ enhances SGI1 transfer through the hybrid T4SS

Attempts to rescue pVCR94ΔX2 Δ*traHG*_C_ with p*traHG*_S_ only partially restored transfer of the mutant plasmid (250-fold reduction of transfer compared to wild-type) (Fig 3), whereas wild-type SGI1ΔIn104 restored transfer of pVCR94ΔX2 Δ*traHG*_C_ to near wild-type level (Fig 4D). This observation suggests that TraN_S_ of SGI1 interacts specifically with the TraHG_S_-containing T4SS. This prompted us to test whether *traN*_S_ could act cooperatively with *traHG*_S_ to enhance the transmissibility of SGI1ΔIn104. We found that although SGI1ΔIn104 Δ*traN*_S_ transfer is not affected by the mutation in the context of wild-type pVCR94ΔX2, the concomitant absence of *traHG*_C_ in pVCR94ΔX2 resulted in a 330-fold decrease of SGI1ΔIn104 Δ*traN*_S_, despite the presence of SGI1ΔIn104-borne *traHG*_S_ and pVCR94ΔX2-borne *traN*_C_ (Fig 4D). In contrast, transfer of pVCR94ΔX2 was not affected in this context. These results showed that all three SGI1 Tra subunits seem to work together to promote its optimal transfer.

### IncC plasmids exert entry exclusion

**Fig 5:**
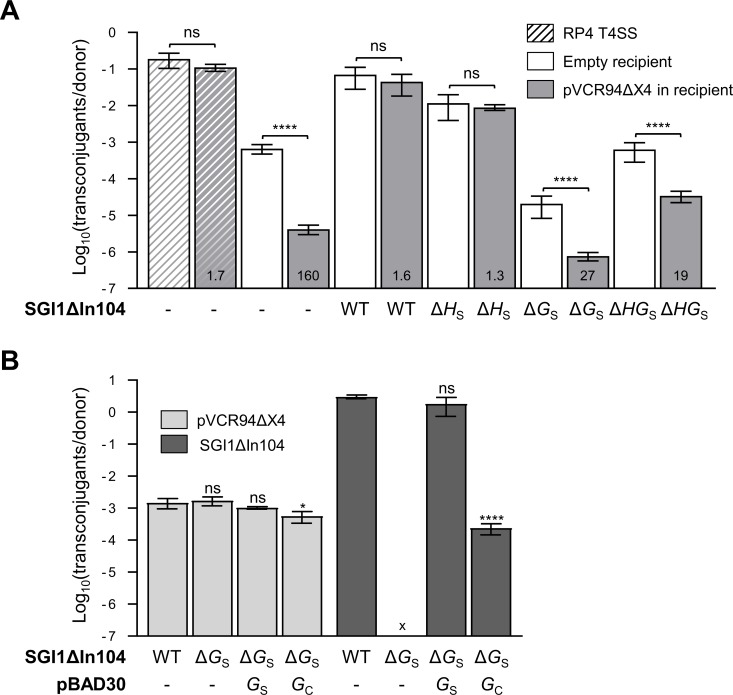
Suppression by *traG*_S_ of IncC entry exclusion. (A) IncC entry exclusion inhibits mobilization of pSU4628. *E*. *coli* SM10λ*pir* (Kn^R^) bearing pSU4628 (Ap^R^) (hatched bars) or *E*. *coli* BW25113 Nx bearing pSU4628 and pVCR94ΔX2 (Sp^R^) in the absence or presence of SGI1ΔIn104 (Kn^R^) or its mutants were used as donors. *E*. *coli* CAG18439 (Tc^R^) bearing or lacking pVCR94ΔX4 (Cm^R^) was used as the recipient. Transfer frequencies are expressed as the number of Tc^R^ Ap^R^ transconjugants per Kn^R^ Ap^R^ donor CFUs (hatched bars) or per Nx^R^ (Kn^R^) Sp^R^ Ap^R^ donor CFUs. Exclusion index (EI) is indicated at the bottom of each gray bar. (B) Effect of *traG*_S_ on mobilization of SGI1ΔIn104 when the helper IncC plasmid is in the recipient. *E*. *coli* BW25113 Nx bearing SGI1ΔIn104 (WT or Δ*traG*_S_, Kn^R^) and *E*. *coli* CAG18439 (Tc^R^) bearing pVCR94ΔX4 (Cm^R^) were crossed. *traG*_S_ and *traG*_C_ complementation assays were carried out with p*traG*_S_ or p*traG*_C_, respectively. Transfer frequencies are expressed as the number of Nx^R^ Cm^R^ transconjugants per Tc^R^ Cm^R^ donor CFUs for pVCR94ΔX4 and Tc^R^ Kn^R^ transconjugants per Nx^R^ Cm^R^ donor CFUs for SGI1ΔIn104. The bars represent the mean and standard deviation values obtained from at least three independent experiments. “x” indicates that the frequency of transfer was below the detection limit (<10^−7^). Statistical analyses were carried out on the logarithm of the values using the one-way ANOVA with Sidak’s post-test (A) to compare each bar to its corresponding control using an empty recipient, and with Tukey’s multiple comparison test (B) for each element. Statistical significance is indicated as followed: ****, *P* < 0.0001; *, *P* < 0.05; ns, not significant.

In F-type T4SSs, TraG is known to be a determinant of entry exclusion in the donor cell [36]. Entry exclusion is a process by which DNA transport from the donor cell is blocked by a recipient cell that contains a plasmid belonging to the same exclusion group. IncA and IncC plasmids seem to have been combined into A/C based on entry exclusion rather than incompatibility [37]. However, whether A/C plasmids exert entry exclusion had yet to be demonstrated. To test this, we monitored the mobilization of pSU4628, a derivative of the broad-host-range mobilizable plasmid CloDF13 (Table 2). Although pSU4628 lacks T4SS genes, it codes for a mobilization protein that recognizes its cognate *oriT* and enables its translocation through P- and F-type T4SSs [38]. As a control, we first tested whether pSU4628 could be efficiently mobilized from *E*. *coli* SM10λ*pir* which bears conjugative plasmid RP4 (P-type) to *E*. *coli* bearing or lacking pVCR94ΔX4. pSU4628 transferred at high frequency regardless of the presence of the IncC plasmid in the recipient (Fig 5A), thereby indicating that IncC plasmids do not exclude entry of DNA mediated by IncP RP4. In contrast, when pVCR94ΔX2 was used to mobilize pSU4628 to the same recipient strains, a 160-fold reduction of transfer (Exclusion index (EI)) was observed if pVCR94ΔX4 was present in the recipient, thereby confirming that IncC plasmids exert entry exclusion.

**Table 2:** Strains and plasmids used in this study.

Strains or element	Relevant genotype or phenotype	Source or reference
*E*. *coli*		
BW25113	F^-^ Δ(*araD-araB*)*567*, Δ*lacZ4787*(::*rrnB-3*), λ^-^, *rph-1*, Δ(*rhaD-rhaB*)*568*, *hsdR514*	[45]
BW25113 Nx	Nx-derivative of BW25113	[11,46]
CAG18439	MG1655 *lacZU118 lacI42*::Tn*10* (Tc)	[47]
SM10λ*pir*	F^-^ *recA*::RP4-2-Tc::Mu λ*pir* (Kn)	[48]
*Plasmids*		
pVCR94ΔX2	Sp-derivative of the IncC plasmid pVCR94 (Su Sp)	[15]
pVCR94ΔX3	Kn-derivative of the IncC plasmid pVCR94 (Su Kn)	[15]
pVCR94ΔX4	Cm-derivative of the IncC plasmid pVCR94 (Su Cm)	This study
pVCR94ΔX2 Δ*traN*_C_	*traN*_C_ deletion mutant of pVCR94ΔX2 (Su Sp)	This study
pVCR94ΔX2 Δ*traG*_C_	*traG*_C_ deletion mutant of pVCR94ΔX2 (Su Sp)	This study
pVCR94ΔX2 Δ*traH*_C_	*traH*_C_ deletion mutant of pVCR94ΔX2 (Su Sp)	This study
pVCR94ΔX2 Δ*traHG*_C_	*traHG*_C_ deletion mutant of pVCR94ΔX2 (Su Sp)	This study
pSIM6	Thermo-inducible expression of λRed recombination (Ts Ap)	[49]
pSIM18	Thermo-inducible expression of λRed recombination (Ts Hy)	[49]
pKD3	Cm template for one-step chromosomal gene inactivation	[45]
pKD13	Kn template for one-step chromosomal gene inactivation	[45]
pCP20	Thermo-inducible expression of Flp recombinase (Ts Ap Cm)	[50]
pBAD30	*ori*_p15A_ *araC P*_BAD_ (Ap)	[51]
p*acaCD*	pBAD30::*acaCD* (Ap)	[15]
p*traN*_C_	pBAD30::*traN*_C_ (Ap)	This study
p*traG*_C_	pBAD30::*traG*_C_ (Ap)	This study
p*traH*_C_	pBAD30::*traH*_C_ (Ap)	This study
p*traN*_S_	pBAD30::*traN*_S_ (Ap)	This study
p*traG*_S_	pBAD30::*traG*_S_ (Ap)	This study
p*traH*_S_	pBAD30::*traH*_S_ (Ap)	This study
p*traHG*_S_	pBAD30::*traHG*_S_ (Ap)	This study
pOP*lacZ*	*oriV*_R6Kγ_; *attP*_λ_; promoterless *lacZ* (Kn)	[15]
pProm*int*	pOP*lacZ P*_*int*_*-lacZ* (Kn)	This study
pProm*xis*	pOP*lacZ P*_*xis*_*-lacZ* (Kn)	This study
pProm*traN*_S_	pOP*lacZ P*_*traN*s_*-lacZ* (Kn)	This study
pProm*traH*_S_	pOP*lacZ P*_*traH*s_*-lacZ* (Kn)	This study
pINT-Ts	*oriR101*; *cI857*; λ*p*_R_-*int*_λ_ (Ap Ts)	[52]
pSU4628	CloDF13::Tn*A*ΔEcoRV (Ap)	[38]
*Genomic islands*		
SGI1	Wild-type SGI1 integrated into the 3’ end of *trmE* (Ap Cm Sp Sm Su Tc)	[15]
SGI1ΔIn104	ΔIn104::*aph*, Kn-derivative of SGI1	This study
SGI1ΔIn104 Δ*traN*_S_	*traN*_S_ deletion mutant of SGI1ΔIn104 (Kn)	This study
SGI1ΔIn104 Δ*traG*_S_	*traG*_S_ deletion mutant of SGI1ΔIn104 (Kn)	This study
SGI1ΔIn104 Δ*traH*_S_	*traH*_S_ deletion mutant of SGI1ΔIn104 (Kn)	This study
SGI1ΔIn104 Δ*traHG*_S_	*traHG*_S_ deletion mutant of SGI1ΔIn104 (Kn)	This study
SGI1ΔIn104 Δ*tra*_S_	*tra*_S_::*cat* deletion mutant of SGI1ΔIn104 (Kn Cm)	This study

Ap, ampicillin; Cm, chloramphenicol; Hy, hygromycin B; Kn, kanamycin; Sm, streptomycin; Sp, spectinomycin; Su, sulfamethoxazole; Tc, tetracycline; Tm, trimethoprim; Ts, thermosensitive.

### TraG_S_ disables entry exclusion between cells bearing IncC plasmids

Genes mediating entry exclusion of A/C plasmids have not yet been characterized; however, by analogy with other F-type T4SSs [36], TraG_C_ is likely the determinant of entry exclusion in donor cells. Since SGI1 codes for its own TraG_S_ subunit, we hypothesized that SGI1 would escape IncC entry exclusion, thereby facilitating DNA exchange between cells bearing IncC plasmids. To test this, we monitored pSU4628 mobilization from a donor bearing both pVCR94ΔX2 and SGI1ΔIn104 to recipient cells bearing or lacking pVCR94ΔX4. pSU4628 mobilization by pVCR94ΔX2 was enhanced by SGI1ΔIn104 at a rate comparable to mobilization by RP4 regardless of the presence of pVCR94ΔX4 in the recipient (EI = 1.6) (Fig 5A), thereby confirming that SGI1 disables IncC entry exclusion. Deletion of *traH*_S_ had no significant impact (EI = 1.3) suggesting it plays no role in disabling IncC entry exclusion. In contrast, when SGI1ΔIn104 Δ*traG*_S_ was used, mobilization of pSU4628 was much reduced and the presence of the IncC plasmid in the recipient resulted in exclusion (EI = 27) (Fig 5A). Mobilization of pSU4628 in the presence of SGI1ΔIn104 Δ*traHG*_S_ was comparable with donors lacking SGI1ΔIn104 (EI = 19).

Recently, Sibor *et al*. [24] showed SGI1 mobilization when the IncC helper plasmid resides in the recipient strain. Since SGI1 is not self-transmissible, this observation suggests that prior to SGI1 mobilization, the donor strain acquires the IncC plasmid, which can then mobilize SGI1 toward the recipient. Such a two-step transfer of SGI1 can only occur if SGI1 disables IncC entry exclusion. We verified this using as a donor *E*. *coli* BW25113 Nx containing either SGI1ΔIn104 (Kn) or its Δ*traG*_S_ mutant, and as a recipient *E*. *coli* CAG18439 bearing pVCR94ΔX4. Selection of intermediate BW25113 Nx transconjugants bearing pVCR94ΔX4 showed that the helper plasmid transferred efficiently regardless of the absence or presence of *traG*_S_ (Fig 5B). Furthermore, while SGI1ΔIn104 transferred at high frequency to CAG18439 with pVCR94ΔX4, we failed to detect transfer of SGI1ΔIn104 Δ*traG*_S_ (Fig 5B). Mobilization was restored to wild-type level when the Δ*traG*_S_ mutant was complemented with *traG*_S_, whereas overexpression of *traG*_C_ partially rescued transfer to levels 4 logs below the wild-type. This confirms that SGI1 fails to transfer to cells harboring an IncC helper if it must rely on a TraG_C_-based T4SS. TraG_S_-based T4SS is critical for SGI1 propagation across bacterial population bearing IncC plasmids.

## Discussion

Most known MGIs are opportunistic passengers riding the T4SS encoded by their conjugative helper element [39]. IncC conjugative plasmids have hitherto been shown to mobilize in *trans* three different MGIs: MGI*Vmi*1 from *V*. *mimicus*, MGI*Vch*Hai6 from *V*. *cholerae* and SGI1 from *S*. *enterica*. Mobilization of both MGI*Vmi*1 and MGI*Vch*Hai6 would rely on the auxiliary mobilization protein MobI, which would play the role of adaptor between the *oriT* of the MGIs and the relaxase of IncC plasmids [15,19,20]. However, this mechanism of mobilization is suboptimal with transfer rates 150 to 200 times lower than the IncC helper plasmid. In strong contrast, SGI1 was reported to transfer more than 10 times better than the same IncC helper plasmid [15,40]. Hence, SGI1 is not merely a free rider of the T4SS encoded by IncC plasmids, but rather tweaks the engine to its own benefit.

This study confirmed the key role of TraN_C_, TraG_C_ and TraH_C_ in the formation of the T4SS of IncC plasmids. Expression of each protein restored the transfer of the corresponding mutant, although it did not enhance its transfer rate above wild-type level. Therefore, unlike the master regulator AcaCD [15], individual production of TraN_C_, TraG_C_ or TraH_C_ is not a limiting step for plasmid transfer. Moreover, our study of the three putative *tra* genes of SGI1 confirmed that not only is expression of these genes AcaCD-dependent as recently shown by Murányi *et al*. [35], but each one also codes for a fully functional T4SS subunit; together the Tra subunits of SGI1 could complement individual *traG*_C_, *traH*_C_ and *traN*_C_ deletion mutants of pVCR94ΔX2. Heterologous complementation of T4SS functions by subunits encoded by different plasmids has already been reported for P-type T4SSs. For instance, the peptide hydrolase TraL from the IncN plasmid pKM101 can replace VirB1 of the VirB T4SS of *Agrobacterium tumefaciens* despite low sequence identity (31%) [41]. Likewise, their TraC and VirB5 components can also be exchanged [42]. Furthermore, the VirB10 homolog TrwE of IncW plasmid R388 can be partially substituted for conjugation by TrwE of *Bartonella tribocorum*, a component of a T4SS involved in pathogenicity [43]. However, such exchanges of T4SS subunits between mobile genetic elements is uncommon and usually prevented in natural systems. For instance, interference between multiple functionally divergent T4SSs that co-occur in *Bartonella* is avoided by tight spatiotemporal regulation of expression or rapid diversification of the T4SS components [44].

**Fig 6:**
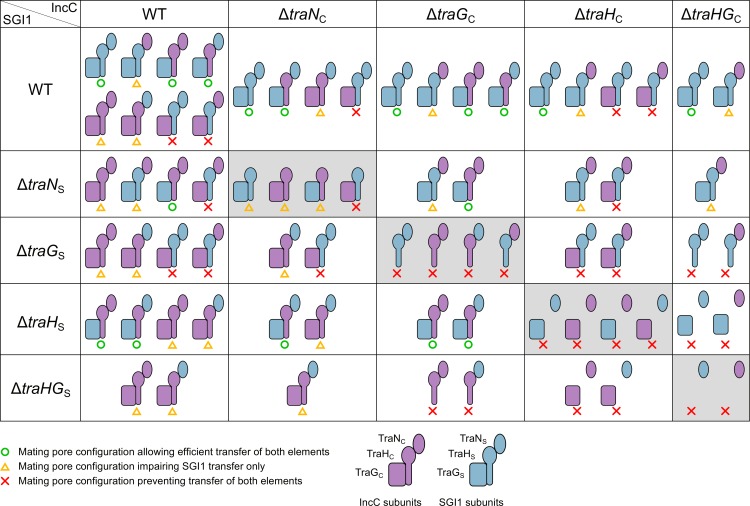
Schematic representation of mating pore configurations and impact on IncC plasmids and SGI1 transfer efficiency. Each mating pore configuration shows only a single subunit of TraN_C/S_, TraG_C/S_, TraH_C/S_, which are represented as described in the figure, and color-coded in purple for IncC plasmid-encoded subunits and blue for SGI1-encoded subunits. The rest of the mating apparatus is not shown and is assumed to be provided by the IncC plasmid. Efficiency of transfer of both elements is indicated by a green circle (optimal or efficient transfer for both elements), an orange triangle (impaired transfer for SGI1 but not IncC plasmid) or a red cross (abolished transfer for both elements) under the corresponding mating pore configuration as inferred from the results (Figs 3 and 4).

Optimal SGI1 transfer depends on which of the subunits are composing the mating pore. Fig 6 illustrates the possible combinations of T4SS subunits and their outcome on SGI1 transfer efficiency inferred from our results (Figs 3 and 4). Our findings challenge a previous report by Kiss *et al*. [28] suggesting that SGI1 *tra*_S_ genes are not involved in SGI1 mobilization. In fact, our results indicate that *traG*_S_ (collaboratively with *traH*_S_ and *traN*_S_) enhances the transfer rate of SGI1ΔIn104 over the helper IncC plasmid (Fig 4). Moreover, *traG*_C_ substitution by *traG*_S_ enables SGI1 to invade cell populations bearing IncC plasmids likely by evading IncC entry exclusion (Fig 5B). Since SGI1 has also been shown to destabilize IncC plasmids [40], this mechanism sheds a new light on the ecological and epidemiological significance of SGI1 and relatives in the propagation of multidrug resistance. In fact, we predict that combination of entry exclusion escaping and IncC plasmid destabilization would result in displacement of IncC plasmids by SGI1 in enterobacterial cell populations bearing IncC plasmids upon contact with a small subpopulation of cell bearing only SGI1.

Deletion of both *traN*_C_ and *traN*_S_ revealed that, despite the lack of putative adhesin that seems to be required for transfer of IncC plasmids, SGI1 can still transfer at low frequency. The TraN_C_/TraN_S_ adhesins, which are thought to stabilize the mating cell pair [18,30,31], are likely required for the transfer of a large DNA molecule such as pVCR94ΔX2 (~120 kb), while the smaller size of SGI1 (~26 kb for SGI1ΔIn104) would render it less vulnerable to premature separation of the mating partners due to the shorter transfer time required to transfer the whole element. In addition, *traN*_C_ and *traN*_S_ could be easily exchanged without drastic impairment of SGI1ΔIn104 or pVCR94ΔX2 transfer. This result is not surprising considering that TraN_C_ and TraN_S_ are the least divergent proteins of the three orthologous pairs (78% identity) (Fig 1, Table 1). An H (TraH_C_ or TraH_S_) and a G (TraG_C_ or TraG_S_) subunit are both required for assembly of a functional mating apparatus. However, we showed that all combinations are not functionally equivalent, as TraH_S_ and TraG_C_ appeared to be incompatible (Fig 4B). Thus, SGI1-encoded subunit TraH_S_ specifically interacts with TraG_S_, strongly enhancing the efficiency of SGI1 transfer. Altogether, our observations suggest that the TraHG_S_ association allows a specific interaction with protein(s) and/or DNA of SGI1 to optimize its transfer. One candidate could be the relaxosome, i.e. the machinery that processes DNA at the SGI1-borne origin of transfer (*oriT*) to allow its transfer. *oriT* of SGI1 and components of the relaxosome that process it remain to be identified and could be partly encoded by SGI1 to confer specificity to the altered mating pore.

Chromosomally integrated SGI1 is not entirely quiescent. While expression of IncC plasmid-borne *tra*_C_ genes strictly depends on AcaCD activation [15], *traHG*_S_ are constitutively transcribed at low level in an AcaCD-independent fashion (Fig 2). Basal expression of *traHG*_S_ strongly suggests that SGI1 primes the bacterial cell to accumulate TraG_S_ and TraH_S_ subunits. While production of these T4SS subunits is likely vain when SGI1 is alone, upon arrival of a helper IncC plasmid, cells primed with TraG_S_ and TraH_S_ might be more prone to rapidly incorporate SGI1-encoded subunits in lieu of IncC-plasmid-encoded ones, thereby favoring the transfer of SGI1 over the helper plasmid. In contrast, expression of *traN*_S_ is strictly dependent upon AcaCD activation (Fig 2) [35]. Tight control over *traN*_S_ expression may have been selected to prevent futile expression of the cell surface-exposed adhesin, which could potentially serve as a receptor for infection by bacteriophages.

In conclusion, unlike any other known mobilizable genomic island described to date, SGI1 not only hijacks the mating pore encoded by IncC plasmids, but also customizes it by inserting its T4SS subunits. This strategy enhances the propagation of SGI1 in bacterial populations as a result of enhanced transfer rates and expansion of its host range to recipient cells bearing IncC plasmids. This study takes us one step further into the comprehension of the intimate relation that links the mobility of unrelated classes of multidrug resistance-conferring mobile genetic elements.

## Materials and methods

### Bacterial strains and media

Bacterial strains and plasmids used in this study are described in Table 2. Strains were routinely grown in lysogeny broth (LB-Miller, EMD) at 37°C in an orbital shaker/incubator and were preserved at -80°C in LB broth containing 15% (vol/vol) glycerol. Antibiotics were used at the following concentrations: ampicillin (Ap), 100 μg/ml; chloramphenicol (Cm), 20 μg/ml; hygromycin B (Hy), 50 μg/ml; kanamycin (Kn), 50 μg/ml or 10 μg/ml for single copy integrants of pOP*lacZ*; nalidixic acid (Nx), 40 μg/ml; spectinomycin (Sp), 50 μg/ml; streptomycin (Sm), 200 μg/ml; sulfamethoxazole (Su), 160 μg/ml; tetracycline (Tc), 12 μg/ml; trimethoprim (Tm), 32 μg/ml. When required, bacterial cultures were supplemented with either 0.02 or 0.2% L-arabinose.

### Mating assays

Conjugation assays were performed by mixing 100 μl of donor cells and 100 μl of recipient cells (typically ~2×10^9^ cells/ml each) that were grown overnight in LB broth at 37°C with suitable antibiotics to ensure retention of the plasmid and SGI1 derivatives. Cells were pelleted by centrifugation for 3 min at 1,200 g, washed once in 200 μl of LB broth and resuspended in 10 μl of LB broth. Mating mixtures were then deposited as drops on LB agar plates and incubated at 37°C for 6 hours. The cells were recovered from the plates in 800 μl of LB broth, vortexed and diluted via serial 10-fold dilutions before plating on LB agar plates containing suitable antibiotics. Donors were selected using a chromosomal marker, and as necessary a marker for pVCR94, SGI1ΔIn104 and/or pSU4628. To induce expression of *tra* genes in complementation assays, mating experiments were carried out onto LB agar plates with 0.02% arabinose. Frequency of transfer was calculated as transconjugants/donor from data obtained from at least 3 parallel mating experiments.

### Molecular biology methods

Plasmid DNA was prepared using the EZ-10 Spin Column Plasmid DNA Minipreps Kit (Bio Basic) according to manufacturer’s instructions. All enzymes used in this study were purchased from New England Biolabs. PCR assays were performed with the primers described in S1 Table. PCR conditions were as follows: (i) 3 min at 94°C; (ii) 30 cycles of 30 sec at 94°C, 30 sec at the appropriate annealing temperature, and 1 minute/kb at 68°C; and (iii) 5 min at 68°C. When necessary, PCR products were purified using an EZ-10 Spin Column PCR Products Purification Kit (Bio Basic) according to manufacturer’s instructions. *E*. *coli* was transformed by electroporation as described by Dower *et al*. [53] in a Bio-Rad GenePulser Xcell apparatus set at 25 μF, 200 V and 1.8 kV using 1-mm gap electroporation cuvettes. Sequencing reactions were performed by the Plateforme de Séquençage et de Génotypage du Centre de Recherche du CHUL (Québec, QC, Canada).

### Plasmid and strain construction

Plasmids and oligonucleotides used in this study are listed in Table 2 and S1 Table. Plasmids used for complementation assays were derived from pBAD30. *traN*_C_, *traG*_C_, and *traH*_C_ were amplified using primer pairs 94traN84EcoRI.for/94traN84EcoRI.rev, 94traG144EcoRI.for/94traG144EcoRI.rev, 94traH143EcoRI.for/94traH143EcoRI.rev, and genomic DNA of *E*. *coli* BW25113 Nx containing pVCR94ΔX2 as the template. Amplicons were digested by EcoRI and cloned into EcoRI-digested pBAD30 using T4 DNA ligase, generating p*traN*_C_, p*traG*_C_ and p*traH*_C_. Likewise, *traN*_S_, *traG*_S_, *traH*_S_ and *traHG*_S_ were amplified using primer pairs SGI105traNSalI.for/SGI105traNSalI.rev, SGI111traGSalI.for/SGI111traGSalI.rev, SGI1s012EcoRI.for/SGI1s012EcoRI.rev, and SGI1s012SalI.for/SGI111traGSalI.rev, and genomic DNA of *E*. *coli* BW25113 Nx containing SGI1 as template. Amplicons were digested by SalI or EcoRI and cloned into SalI or EcoRI-digested pBAD30 using the T4 DNA ligase, generating p*traN*_S_, p*traG*_S_, p*traH*_S_ and p*traHG*_S_.

PCR fragments containing the promoter region upstream of *int*, *xis*, *traN*_S_, *traHG*_S_ were amplified using primer pairs SGI1promintPstI.for/SGI1promintPstI.rev, SGI1promxisPstI.for/SGI1promxisPstI.rev, SGI1promtraNPstI.for/SGI1promtraNPstI.rev, SGI1promtraHPstI.for/SGI1promtraHPstI.rev and cloned into the PstI restriction site of pOP*lacZ* to produce pProm*int*, pProm*xis*, pProm*traN*_S_, pProm*traH*_S_, respectively [15]. The resulting plasmids were verified by restriction profiling and DNA sequencing. These vectors were integrated in single copy into the chromosomal site a*ttB*_λ_ of *E*. *coli* BW25113 Nx using pINT-Ts [52].

Deletion mutants of pVCR94ΔX2 and SGI1 were constructed using the one-step chromosomal gene inactivation technique with pSIM6 or pSIM18 (Table 2) [45]. For pVCR94ΔX2, deletions of *traN*_C_, *traG*_C_, *traH*_C_ and *traHG*_C_ were obtained using primer pairs, 94del84traN.for/94del84traN.rev, 94del144traG.for/94del144traG.rev, 94del143traH.for/94del143traH.rev, 94del143traH.for/94del144traG.rev, respectively, and pKD3 as the template (Table 2 and S1 Table). SGI1 derivative SGI1ΔIn104 was obtained using primer pair SGI1delVar.for/SGI1delVar.rev and pKD13 as the template. Subsequent deletions of *traN*_S_, *traG*_S_, *traH*_S_, *traHG*_S_ and *traN*_S_-*traH*_S_ region in SGI1ΔIn104 were obtained using primer pairs, SGI1delS005.for/SGI1del05traN.rev, SGI1delS011.for/SGI1delS011.rev, SGI1delS012.for/SGI1delS012.rev, SGI1delS012.for/SGI1delS011.rev and SGI1delS012.for/SGI1del05traN.rev, respectively, and pKD3 as the template. Substitution of the *aph* (Kn) resistance gene with the *cat* (Cm) resistance gene in pVCR94ΔX3 was carried out using the same approach with primers 94DelXnoFRTcm.for and 94DelXnoFRTcm.rev, and pKD3 as the template, yielding pVCR94ΔX4.

When possible, the antibiotic resistance cassette was removed from the resulting construction by Flp-catalyzed excision using the pCP20 vector [50]. All deletions were verified by PCR and antibiotic resistance profiling.

### β-galactosidase assays

Qualitative assays on solid LB agar plate were done using 40 μg/ml 5-bromo-4-chloro-3-indolyl-β-D-galactopyranoside (X-gal) as the substrate with or without 0.02% arabinose. Plates were observed after overnight incubation at 37°C.

Quantitative liquid assays using o-2-nitrophenyl-β-D-galactopyranoside (ONPG) as the substrate were done according to a protocol adapted from Miller [54]. After overnight incubation at 37°C in 4 ml LB broth supplemented with appropriate antibiotics, cultures were refreshed 1:100 in 4 ml LB broth supplemented with 10 μg/ml kanamycin, 25 μg/ml ampicillin and 0.2% arabinose except for the non-induced controls. Cultures were incubated for 5 hours at 37°C with shaking prior to sampling for enzymatic assays. OD measurements for enzymatic assays were performed using a Multiskan Go Microplate Spectrophotometer (Thermo Scientific). Each experiment was performed in at least three independent biological replicates. Induction ratios were calculated by dividing the “induced” values by the “non-induced” values whereas the control ratios were calculated by dividing the “non-induced” values by the control values.

## Supporting information

S1 Fig Effect of *tra*C/S genes on cotransfer of IncC plasmids and SGI1. Effect of *traG*_C_ and *traG*_S_ (A), and *traH*_C_ and *traH*_S_ (B) and *traN*_C_ and *traN*_S_ (C) and combinatory mutants (D), on cotransfer of pVCR94ΔX2 and SGI1ΔIn104. For details, refer to legend of Fig 3.
(PDF)

S1 Table Primers used in this study. (PDF)
