# Supplementary figures and images for: Salmonella genomic island 1 (SGI1) reshapes the mating apparatus of IncC conjugative plasmids to promote self-propagation
Source: PLoS Genet. 2017 Mar 29;13(3):e1006705. doi: 10.1371/journal.pgen.1006705 (PMC5389848; doi:10.1371/journal.pgen.1006705)

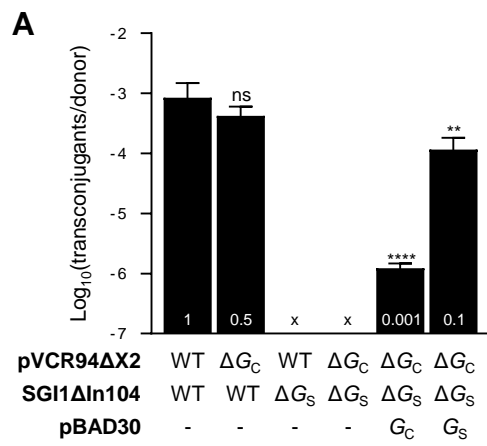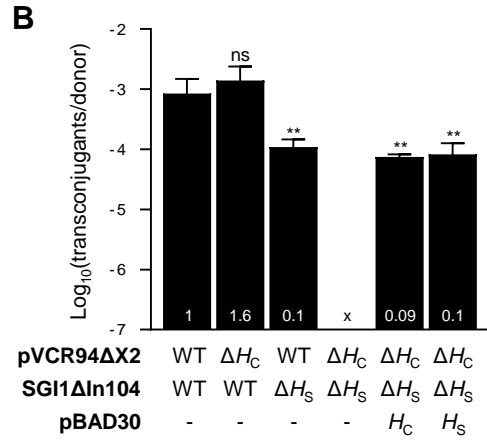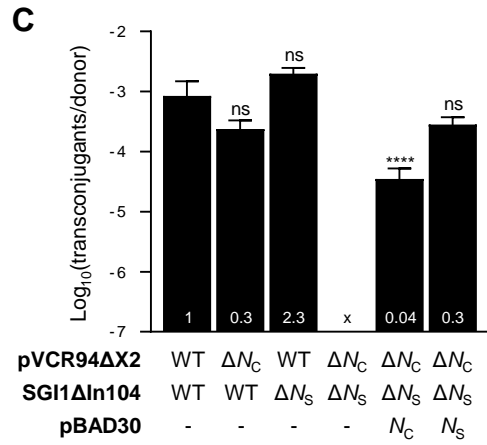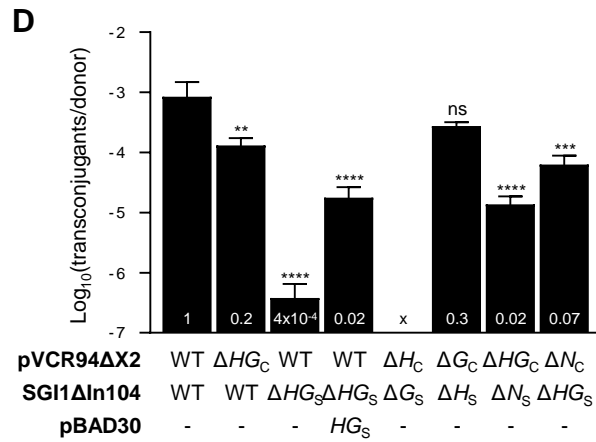

Supplement: S1 Fig — Effect of traGC and traGS (A), and traHC and traHS (B) and traNC and traNS (C) and combinatory mutants (D), on cotransfer of pVCR94ΔX2 and SGI1ΔIn104. For details, refer to legend of Fig 3. (PDF) [file pgen.1006705.s001.pdf]
