# Supplementary material for: Salmonella genomic island 1 (SGI1) reshapes the mating apparatus of IncC conjugative plasmids to promote self-propagation
Source: PLoS Genet. 2017 Mar 29;13(3):e1006705. doi: 10.1371/journal.pgen.1006705 (PMC5389848; doi:10.1371/journal.pgen.1006705)
Supplement: S1 Table — (PDF) [file pgen.1006705.s002.pdf]

S1 Table. Primers used in this study

Name	Nucleotide sequence (5’ to 3’)[a]
94traN84EcoRI.for	NNNNNNGAATTCAAGGAGGAATAATAAATGCGAAGTCATAATTACTTTATGA
94traN84EcoRI.rev	NNNNNNGAATTCTTATCCACCACCACCAGTTGGCGCG
94traG144EcoRI.for	NNNNNNGAATTCAAGGAGGAATAATAAATGGGATCTTTTTCAATCCACTCTA
94traG144EcoRI.rev	NNNNNNGAATTCTTAGAACCTTTGATTTGCTATGTT
94traH143EcoRI.for	NNNNNNGAATTCAAGGAGGAATAATAAATGGTCACGCACAAGACATTAAAA
94traH143EcoRI.rev	NNNNNNGAATTCTTAGTTCGTGCTAGGAGGATTGGA
SGI105traNSalI.for	NNNNNNGTCGACAAGGAGGAATAATAAATGTCCACTATGCCGCCCAT
SGI105traNSalI.rev	NNNNNNGTCGACTCAACCACCATTGCCATCCAA
SGI111traGSalI.for	NNNNNNGTCGACAAGGAGGAATAATAAATGGATTTTAGTATTTATTCCGT
SGI111traGSalI.rev	NNNNNNGTCGACTTAACGGCCTCCTTTGGCTA
SGI1s012EcoRI.for	NNNNNNGAATTCAAGGAGGAATAATAAATGAGAGTCTCTCCCCCTTA
SGI1s012EcoRI.rev	NNNNNNGAATTCTTACTCCTTTTTCCCTGATGCTT
SGI1s012SalI.for	NNNNNNGTCGACAAGGAGGAATAATAAATGAGAGTCTCTCCCCCTTA
SGI1promintPstI.for	NNNNNNCTGCAGACACCTTGAGCAGGGCAAA
SGI1promintPstI.rev	NNNNNNCTGCAGCGTTACTCCAAAATTTTAACT
SGI1promxisPstI.for	NNNNNNCTGCAGGTTATTGATAGACCTAGTTTAT
SGI1promxisPstI.rev	NNNNNNCTGCAGGGCCAATGTGCCGGTTT
SGI1promtraNPstI.for	NNNNNNCTGCAGCCAGCTTTTTAGTTTGGATA
SGI1promtraNPstI.rev	NNNNNNCTGCAGGATAGAGCATTGCGAGCAAT
SGI1promtraHPstI.for	NNNNNNCTGCAGTAGGTTTCGTGTCACCCGAA
SGI1promtraHPstI.rev	NNNNNNCTGCAGTTAAAGCTCCTCTTTTAGAA
94del84traN.for	TCTTCTCAGATAGCATGGGAAGGTTGAAATGGAGAACACAGTGTAGGCTGGAGCTGCTTC
94del84traN.rev	AATCAATCTACATGAGAAAGGGGCCGAAAGGCCCCTTTTTTTATTACATATGAATATCCTCCTTA
94del144traG.for	TTGTCCAATCCTCCTAGCACGAACTAAGGAGCTATTGGAGTGTAGGCTGGAGCTGCTTC
94del144traG.rev	TAAGGGGGCCTGCTGGCCCCCTTATTATTTGGTTGGTCTTTTATTACATATGAATATCCTCCTTA
94del143traH.for	CTCATGAAGTACATCCGGGACAAATTACAGGAGAACTAAGGTGTAGGCTGGAGCTGCTTC
94del143traH.rev	GAAAAGCAGAGTCACCGATAGAGTGGATTGAAAAAGATCCCATATGAATATCCTCCTTA
SGI1delVar.for	TATTTTAACATGTATATAGATTAAATTCCAATCAACTTGTTCGGAATAGGAACTTCAAGA
SGI1delVar.rev	CAAACCACCATTTTCTTAACAGTTCATAACTTAGCAAGTTAGAGCGCTTTTGAAGCTCA
SGI1delS005.for	CGACATAGCATTATCCAAACTAAAAAGCTGGAGAAATGCTGTGTAGGCTGGAGCTGCTTC
SGI1del05traN.rev	ACAAAAACTTTCACATGTGAAAGTTTCTCATTGATAAACATCATTACATATGAATATCCTCCTTA
SGI1delS011.for	TTTACTTGCGGCCCAAGCATCAGGGAAAAAGGAGTAACTAGTGTAGGCTGGAGCTGCTTC
SGI1delS011.rev	TAAGCTCTTTAATTGCGCGTCTATATTCAGCTCGTTTGCTCATATGAATATCCTCCTTA
SGI1delS012.for	GTAGGCTTTCGGGTGACACGAAACCTATTGGAGCAACAGTGTGTAGGCTGGAGCTGCTTC
SGI1delS012.rev	CTAGAAATGCGGCATCACCGACGGAATAAATACTAAAATCCATATGAATATCCTCCTTA
94DelXnoFRTcm.for	GGGTAGAATAAGCCTCGATATAGTCATGTGACTAAAAGGCGGAATAGGAACTTCATTTA
94DelXnoFRTcm.rev	TCGTTAACTGCACATTCGGGATATTTCTCTATATTCGCGGGCCTACCTGTGACGGAAGAT

[a] restriction sites are underlined
